# The use of nanomaterials in advancing photodynamic therapy (PDT) for deep-seated tumors and synergy with radiotherapy

**DOI:** 10.3389/fbioe.2023.1250804

**Published:** 2023-10-02

**Authors:** Deepak Dinakaran, Brian C. Wilson

**Affiliations:** ^1^ National Cancer Institute, National Institute of Health, Bethesda, MD, United States; ^2^ Radiation Oncology, Sunnybrook Health Sciences Centre, University of Toronto, Toronto, ON, Canada; ^3^ Princess Margaret Cancer Centre, University Health Network, University of Toronto, Toronto, ON, Canada

**Keywords:** radiation, photodynamic therapy (PDT), radioPDT, X-ray PDT, XPDT, immune activation

## Abstract

Photodynamic therapy (PDT) has been under development for at least 40 years. Multiple studies have demonstrated significant anti-tumor efficacy with limited toxicity concerns. PDT was expected to become a major new therapeutic option in treating localized cancer. However, despite a shifting focus in oncology to aggressive local therapies, PDT has not to date gained widespread acceptance as a standard-of-care option. A major factor is the technical challenge of treating deep-seated and large tumors, due to the limited penetration and variability of the activating light in tissue. Poor tumor selectivity of PDT sensitizers has been problematic for many applications. Attempts to mitigate these limitations with the use of multiple interstitial fiberoptic catheters to deliver the light, new generations of photosensitizer with longer-wavelength activation, oxygen independence and better tumor specificity, as well as improved dosimetry and treatment planning are starting to show encouraging results. Nanomaterials used either as photosensitizers *per se* or to improve delivery of molecular photosensitizers is an emerging area of research. PDT can also benefit radiotherapy patients due to its complementary and potentially synergistic mechanisms-of-action, ability to treat radioresistant tumors and upregulation of anti-tumoral immune effects. Furthermore, recent advances may allow ionizing radiation energy, including high-energy X-rays, to replace external light sources, opening a novel therapeutic strategy (radioPDT), which is facilitated by novel nanomaterials. This may provide the best of both worlds by combining the precise targeting and treatment depth/volume capabilities of radiation therapy with the high therapeutic index and biological advantages of PDT, without increasing toxicities. Achieving this, however, will require novel agents, primarily developed with nanomaterials. This is under active investigation by many research groups using different approaches.

## Introduction

Cancer research is crowded with therapeutic options that promise higher efficacy with less toxicity. Several new targeted biologic agents intended to combat tumor directly or indirectly via modulation of the immune or microvascular systems were predicted to be paradigm-shifting (Baudino, 2015) and have led some to question if there is a future role for traditional therapies such as radiation, surgery and chemotherapy (Arruebo et al., 2011). However, while clinical trials with some of these agents have demonstrated promise, many have failed (Mallarkey and Coombes, 2013; Maeda and Khatami, 2018; Sabnis and Bivona, 2019; Wong et al., 2019). In comparison, renewed interest in aggressive local therapies are leading to more radical surgeries and stereotactic radiotherapy for loco-regional disease (Weichselbaum and Hellman, 2011; Cheung et al., 2019). Even in the metastatic setting, surgical excision and stereotactic radiotherapy for treating the primary tumor bulk have demonstrated significantly increased progression-free and overall survival (Iyengar et al., 2018; Gomez et al., 2019; Palma et al., 2019). These benefits also exist with more advanced targeted drug therapies and perhaps may even synergistically, e.g., immunotherapy agents, for better overall treatment outcomes (Demaria et al., 2016; Weichselbaum et al., 2017), especially using localized therapies with potential abscopal effects.

A significant continuing challenge is to limit the toxicities of these aggressive local therapies. Surgical oncology has made significant advances in recent decades through improved and minimally-invasive techniques (Chang and Rattner, 2019). Further advances may arise through robotic-assisted surgeries but, despite many years of use, this has yet to translate into clear clinical benefit (Seigne et al., 2019). Combination therapies, including neoadjuvant and adjuvant chemotherapy, have demonstrated benefit in advanced and difficult-to-treat tumors (Forbes, 1982). Factors in individual patients that limit more aggressive management include comorbidities, advanced age, disease-related decline in performance status and long-term side effects from prior cancer therapies.

Radiotherapy has made great advances in local dose escalation without undue toxicities, mainly through advances in technologies for dose delivery. High-precision radiotherapy has existed for some time in machines such as the Gamma Knife, Cyber Knife, Tomotherapy, and linear accelerators (LINACs) with motion-tracking and beam modulation. The addition of inverse planning-based intensity-modulated radiotherapy (IMRT) has also advanced the field. These technologies allow high spatial precision that can rival surgery with less acute toxicity (Chang et al., 2015). This has proven useful in managing primary as well as oligometastatic disease (Palma et al., 2019). Clinical implementation has hit its stride in the past decade but added benefits of further refinements in dose delivery may be limited. Thus, we are approaching sub-millimeter expansion for planning target volumes (PTVs), whereby the area under treatment is intentionally expanded beyond the known clinical disease to accommodate for motion and setup error, but every last mm and fraction of a mm gained will likely yield diminishing clinically significant returns. New modalities such as ultra-high dose-rate FLASH radiotherapy may provide some additional benefit in maximizing tumor treatment efficacy while minimizing toxicity (Bourhis et al., 2019) but this remains to be proven. Further refinements in radiation dosing and fractionation, and novel clinical applications such as in oligometastatic disease may continue to advance the field but, in the absence of newer methods of safe dose escalation, we will eventually reach the limits of contemporary radiotherapy.

A variety of other biophysical modalities for treating cancers locally have been developed in the past few decades to improve the therapeutic index and deliver higher treatment efficacy with lower toxicity. Techniques such as radiofrequency ablation (RFA), high-intensity focused ultrasound (HIFU), endovascular embolization, hyperthermal therapy and cryotherapy have aimed to increase local tumor control and, in some cases, also possibly generate an immunologic response (Goode and Matson, 2004; Glazer and Curley, 2011; Nishikawa and Osaki, 2013). These various techniques have met with different degrees of success: some have established new niches but none have been widely transformative to date. Their limitations, including the invasiveness, lack of intrinsic tumor specificity and collateral damage to surrounding normal tissue, have limited their applications.

One form of local therapy that has demonstrated high anti-tumor activity with low toxicity is photodynamic therapy (PDT). First described over 100 years ago, this modality re-emerged in the early 1980s, driven by the availability of lasers as activating light sources and by the development of potent photosensitizers. The first regulatory approval, in 1993 in Canada, was for treatment of refractory superficial bladder cancer (van Straten et al., 2017) and PDT has since been approved in multiple countries for a range of tumor types and stages; from curative treatment of premalignant lesions to palliation of advanced disease. In its early modern phase, PDT was widely projected to be the next paradigm shift in local cancer management (Santos et al., 2019). Currently, it is well accepted for treating superficial cancers, including endoscopically-accessible lesions (Santos et al., 2019). It has also proven to be effective for tumors that are refractory to radiotherapy (Hatogai et al., 2016). However, PDT has failed to gain broader acceptance. A major technical factor is the difficulty of using PDT for deep-seated and larger solid tumors, due primarily to the limited penetration of the red/near-infrared activating light: in the simplest case of external light source irradiation or interstitial fiberoptic light sources, effective treatment depths or radii of 5–10 mm are typical. This is further compounded by these deeper penetrating, but longer, wavelength photons possessing lower amounts of energy needed for the photochemical reaction to generate singlet, with the theoretical maximum wavelength being about 800 nm to successfully generate singlet oxygen. Some of the most common cancers, such as lung, prostate, breast and gastrointestinal, are mainly deep-seated and are difficult or at least technically complex to treat for complete tumor destruction (Siegel et al., 2019). This limitation may be changing and eventually allow effective treatment anywhere in the body. One such evolution involves overlap with radiation oncology, whereby the precise targeting of modern X-ray technologies may provide the excitation energy to effect PDT (Ren et al., 2018; Cline et al., 2019), as discussed below.

## PDT mechanisms and technologies

PDT uses a two-step activation process to induce cytotoxic tissue damage. Visible or near-infrared (NIR) light provides the energy to activate photosensitizing molecules (PS), thereby generating cytotoxic reactive oxygen species (ROS) (Debele et al., 2015). The light and photosensitizer alone are each essentially non-toxic with no therapeutic activity. There is minimal heat generated in the tissue, distinguishing PDT form photothermal therapy where higher-power (laser) light is used to destroy tumor tissue through photocoagulation. However, as recently discussed by Wilson and Weersink (Wilson and Weersink, 2020), these two modalities do share many common technical features and the optimal choice between them can depend on the tumor size and location.

The most commonly used PSs in patients are porphyrin ring-based molecules that were “re-discovered” in the 1960s, following a much longer history as both phototherapeutic and photodiagnostic (fluorescence imaging) agents (Castano et al., 2004). They are analogous to endogenous porphyrin ring structures that are precursors in the heme biosynthetic pathway (Castano et al., 2004). This molecular structure confers important photophysical and photochemical; properties that make them efficient PDT agents. The key structure is a tetrapyrrole backbone that is also found in chlorins and phthalocyanines. The photodynamic and pharmacokinetic properties may be “fine-tuned” by the altering the specific molecular structure (Zhang et al., 2018).

The most commonly used light sources for clinical PDT are either diode lasers or light emitting diodes (LEDs): the former allows efficient light delivery through small diameter (typically, 200–400 μm) optical fibers for endoscopic or interstitial use, while the latter are relatively inexpensive and can be configured in different shapes and sizes to treat different tumor sites. Wavelength-filtered lamp-based systems are also used for skin malignancies or, more commonly, benign skin conditions (Brancaleon and Moseley, 2002). These various light sources produce energy continuously (CW) and treatments typically take minutes to tens of minutes to deliver sufficient local energy density (∼100 J/cm^2^) at a low enough lower power density (<∼100 mW/cm^2^) to avoid tissue heating and/or photochemical depletion of tissue oxygenation. The wavelength used in most clinical trials to date are in the 630–730 nm range to achieve usable tissue penetration while still providing sufficient photon energy to activate the photosensitizer. For more superficial lesions (e.g., skin, bladder), blue or green light are also used. Preclinically, ultrashort NIR laser pulses have been investigated to perform 2-photon activation, which may provide somewhat deeper tissue penetration and, more importantly, allow very precise (diffraction-limited) control over the three-dimensional activation volume. This comes with the drawback that 2-photon PDT needs a specific photosensitizer with a good absorption cross-section to both wavelengths and the specific molecular structure to convert their energies into singlet oxygen. The potential applications have been mainly in ophthalmology and dermatology (Wan et al., 2016; Bolze et al., 2017a): one report (Bolze et al., 2017b) has suggested effective treatment depths of several cm can be achieved in solid tumor, but the interpretation of these results is controversial. Ultrasound has also been investigated as the means to activate PSs (sonoluminescence) but the underlying biophysical mechanisms are not well understood and it has proven difficult to make this a robust approach (Wan et al., 2016; Bolze et al., 2017a).

**FIGURE 1:**
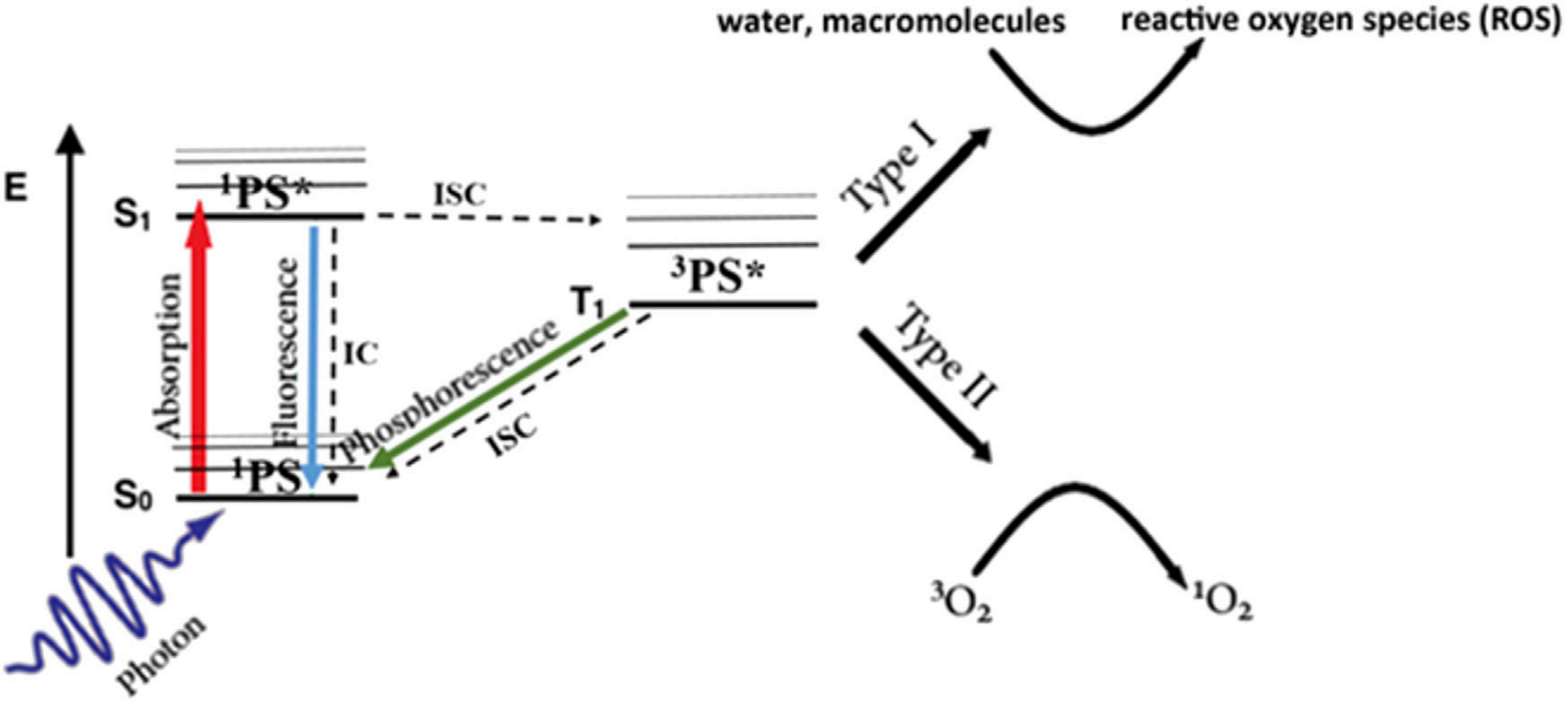
Simplified Jablonski energy diagram of photodynamic activation. Radiative transitions are shown as colored arrows and non-radiative transitions as black/dashed arrows.

As illustrated in Figure 1, upon absorption of a photon of suitable wavelength (energy), the ground-state photosensitizer, ^1^PS (S_0_), is excited to an electronic singlet state, ^1^PS* (S_1_). This can return to the ground state either via non-radiative internal conversion or by fluorescence emission, which is widely used for diagnostics and image-guided surgery (Daneshmand et al., 2018a; Ottolino-Perry et al., 2021; Sutton et al., 2023), or it can undergo intersystem crossing to a triplet state, ^3^PS^*^ (T_1_) (Castano et al., 2004). This can decay back to the ground state either non-radiatively or by phosphorescence emission, or by reacting with a suitable molecular substrate via so-called Type I or Type II pathways. In Type I the triplet state reacts directly with biomolecules such as lipid membranes or water to form free radical species (Castano et al., 2004). In the Type II pathway, the excess energy is transferred to ground-state molecular oxygen, ^3^O_2_, to form highly-reactive singlet oxygen, ^1^O_2_. The Type II pathway is believed to be the dominant process with most (Castano et al., 2005a) but not all (Neuschmelting et al., 2018) clinical photosensitizers. One consequence is that the efficacy depends on the available molecular oxygen in the local tumor environment (see below). As a result of its high reactivity, the lifetime of ^1^O_2_ in cells/tissues is short (<1 μs), so that it diffuses only tens of nm before interacting and causing damage. Hence, the subcellular localization of the PS is critical in determining the organelle(s) damaged and, thereby, the resulting cell-death pathway and level of cytotoxicity: for example, the same amount of singlet oxygen is ∼10-fold more cytotoxic if the PS is localized to mitochondria rather than the plasma membrane (Mahalingam et al., 2018). This confers a high degree of biological control. For example, if the treatment light is delivered while the PS is still in the circulation, then vascular endothelial cell death and ischemia will be the primary route of tumor destruction rather than direct killing of the tumor cells. This has been exploited in some clinical studies (Azzouzi et al., 2017). The use of oxygen-independent photosensitizers is also an option (Larue et al., 2021), although this does not circumvent the need to deliver the PS, which may be compromised in poorly-vascularized regions of tumor.

The net effect of PDT is mainly to promote oxidative stress, which manifests in different processes, at the level of cellular, tumor and immune-system responses. In the cancer cell, the ^1^O_2_ can react with macromolecules, particularly lipids. Depending on the subcellular localization of the PS, this leads to plasma membrane damage and/or disruption of lysosomes, mitochondria or endoplasmic reticulum, inducing stress responses that lead to cell necrosis, apoptosis or autophagy (Castano et al., 2005a). Similar processes may occur in vascular endothelial cells (Castano et al., 2005b). The dying cells also release cytokine signals that promote death of nearby cells (the bystander effect) (Wang et al., 2018). Lastly, an important contributor to PDT’s clinical efficacy is its ability to “prime” the immune system, an effect that was observed initially in preclinical murine studies in which PDT generated durable cures in immune-competent animals (Korbelik et al., 1996), but has also been observed in patients (Thong et al., 2007; Thong et al., 2008; Beltrán Hernández et al., 2020).

It is well established that PDT-mediated damage via lipid peroxidation of the plasma membrane and cell organelles elicit strong pro-inflammatory signalling and results in a robust innate immune response leading to immunological cell death of cancer cells (Castano et al., 2005a). This also potentiates an adaptive response for long-term control. The PDT oxidative stress causes vascular injury-induced tumor ischemia, as well as strong direct cytotoxicity via lipid membrane disruption, cell death and spillage of cytoplasmic contents that act as damage-associated molecular patterns (DAMPs) to activate multiple signaling pathways such as Toll-like receptors (TLRs) and RIG-I-like receptors (RLRs) on dendritic cells, heat shock protein (HSP) pathways, nuclear factor kappa B (NFkB), tumor necrosis factor alpha (TNFα) and activator protein 1 (AP-1). DAMPs also activate CD8^+^ cytotoxic T lymphocytes through similar mechanisms as in radiotherapy, leading to multiple pro-inflammatory interleukins, chemokines, interferons and macrophage inflammatory proteins (MIP). In turn, the innate and adaptive immune response activates T lymphocytes, macrophages, natural killer (NK) cells and neutrophil infiltration to kill tumor cells directly, and also produces further pro-inflammatory activity, immune cell activating mediators and complement cascade activation (Panzarini et al., 2013; Chen et al., 2017a). Overall then, an acute and powerful inflammatory response and induction of early and late immune responses mediates the potential abscopal effects of PDT that have been seen in a range of preclinical and clinical studies (Moore et al., 1993; Castano et al., 2005b; Chen and Zhang, 2006; Takahashi and Misawa, 2007; Liu et al., 2008; Zou et al., 2014; Debele et al., 2015; Retif et al., 2015). These effects are recognized increasingly as significantly contributing to the efficacy of PDT, in terms of both primary tumor response and reduced tumor progression and metastatic risk and represent a clear advantage over other local biophysical therapies.

Ionizing radiation has also been investigated to potentiate immunotherapy drugs, particularly in the scenario of poor response to immune checkpoint inhibition (Panzarini et al., 2013; Weichselbaum et al., 2017). The main proposed mechanisms are via DAMPs in the microenvironment that lead to pro-inflammatory cytokines such as interleukin-1β (IL-1β), transforming growth factor β (TGF-β), fibroblast growth factor (FGF) and TNFα. In addition, cytosolic DNA ingested by dendritic cells from radiation-damaged tumor cells produce cyclic guanine monophosphate-adenosine monophosphate (cGAMP) via cGAMP synthase (cGAS) and stimulate interferon genes (STING) to transcribe type I interferon (IFN). ICD dampening simultaneously exists through TGF-β and IL-10 mediated regulatory T lymphocytes (T_reg_) and myeloid-derived suppressor cell (MDSC) activation, which act to suppress cytotoxic T lymphocyte activity (Vanpouille-Box et al., 2017). These competing ICD promoting and dampening pathways with radiotherapy can lead to a spectrum of responses such as tumor elimination, equilibrium, dormancy or escape. In practice, the clinical response to combined radiotherapy and immunotherapy has not been conclusively positive (Kwon et al., 2014; Asna et al., 2018).

Some of the immune-priming mechanisms are shared between radiotherapy and PDT, but key differences do exist. Both can generate abscopal effects in isolation via systemic spread of T and B lymphocytes sensitized to tumor neoantigens. Both rely on DAMP-mediated antigen-presenting cell (APC) activity, leading to recruitment and activation of effector T lymphocytes (T_eff_), and both cause chemokine and chemotaxin release to promote T lymphocyte infiltration and inflammation (Panzarini et al., 2013; Weichselbaum et al., 2017). A key feature of PDT is that it can elicit a strong innate immune response causing a high degree of tumor inflammation that tips the balance of immune-priming and immune-dampening towards the former. ROS-mediated damage to tumor cells favours necrosis and a high load of DAMPs from the cytosol being directly released into the extracellular environment. This induces strong cytokine/chemokine activation for the innate immune response and produces many neoantigens for the adaptive immune response.

In radiation therapy DNA-mediated cell death, together with DAMP presentation and cGAS-STING activation, does not produce as profound inflammatory and innate immune responses as PDT (Lee et al., 2009; Chen et al., 2017a) that contribute to the clinical outcomes. We note also that there is evidence of a negative immune response from surgical resection of tumors, both in animal models and in patients (Salo, 1992; Onuma et al., 2020), so that using PDT in the adjuvant setting with radiotherapy and/or surgery may be advantageous. Interestingly, the immune-stimulating profile of PDT and radiotherapy have not been directly compared to our knowledge, although the expectation is that PDT will be the more powerful immune stimulator given its central role in clinical PDT and the known mechanism of action.

Mechanistically, the cellular and, hence, tumor response to PDT is much more rapid (hours-days) than radiotherapy, since it depends on killing the tumor cells rather than reducing their proliferative capacity. This is due primarily to the initial targets for PDT damage being extra-nuclear, which accounts also for the extremely low level of induced resistance observed with PDT (Desmettre et al., 2004; Casas et al., 2011) and the fact that PDT is effective even in radiation-resistant tumors (Stewart et al., 1998a; Oniszczuk et al., 2016). It is known, however, that active targeting of photosensitizer to the cell nuclear can increase tumor cell kill by more than 1,000-fold (Long et al., 2022), although this has not been exploited clinically to data.

The tumor response to PDT may exceed what the direct, short-range ROS-mediated cancer cell killing alone would predict (Castano et al., 2005a). As indicated above, bystander and induced immunological effects can induce wider cell death via the above cell signalling factors (Castano et al., 2005a). Normal tissue is more resistant than tumors to these secondary indirect effects. Hence, in addition to preferential PS uptake/retention and geometric confinement of the treatment light, this contributes to the high cancer selectivity of PDT (Castano et al., 2005a). These indirect effects have parallels to spatially-fractionated radiotherapy, a modality that focuses high-dose radiation in a grid-like pattern on the target achieving tumor responses with less acute radiotoxicity. However, this has major logistical challenges and the efficacy is still limited by radioresistance and long-term dose-related toxicity (Billena and Khan, 2019).

A key difference between PDT and radiotherapy is the toxicity profile of each. The short-term toxicities with PDT have related primarily to skin photosensitivity. With the first-generation PSs such as Photofrin this required that the patient avoid direct sunlight and bright artificial light for several weeks. This issue is much less severe with current PSs for which the skin photosensitivity is short lived, requiring precautions for typically only a few days. Photosensitizers also have minimal systemic toxicities that are easily managed or avoided. Local pain during light treatment has been reported in skin lesions with aminolaevulinic acid-PPIX (ALA-PPIX) mediated PDT but is transient and can be managed by local cooling, analgesics or other conservative measures (Warren et al., 2009). As far as is known, PDT has no significant long-term toxicity, which contrast with radiotherapy where both acute and late off-target effects are seen, including fibrosis, lymphatic damage, organ dysfunction and increased rate of secondary malignancies.

A further advantage of PDT is that the normal host tissues show excellent healing responses, due to the non-thermal nature of PDT avoiding damage to collagen that preserves the tissue architecture. This is obviously important in treating skin lesions for good cosmesis but is also critical in treating tumors of hollow organs in order to maintain the organ integrity. There is also evidence that PDT is nerve sparing, which is highly relevant in sites such as the head and neck and prostate (Wright et al., 2006). Finally, while PDT is usually administered as a single acute dose, in the event of incomplete tumor response or local recurrence after treatment, it can be repeated without loss of efficacy or increased risk to achieve complete responses: this has been demonstrated, for example, in prostate cancer that is recurrent after radiotherapy (Nathan et al., 2002). Repeat treatment may involve either re-administration of both PS and light or, with PSs having long circulation/residence times, light only.

## PDT in clinical cancer care

The modern era of PDT in clinical cancer therapy started in 1993 when a PPIX derivative called Porfimer sodium (Photofrin) gained regulatory approval in Canada for use in refractory superficial bladder cancer (van Straten et al., 2017). Approvals followed for several other PSs in many different countries. To date several hundred trials have been conducted with PDT, the majority of which showed positive outcomes (Santos et al., 2019). These trials have included PDT as monotherapy and in combination with radiation, surgery, chemotherapy or, more recently, targeted drug therapy. The oncological indications have included primary and recurrent cancers in the bladder, skin (mainly basal cell carcinoma), eye, lung, pancreas, head and neck, esophagus, stomach, biliary canal, anal canal, brain, breast, cervix and prostate. Metastatic disease has generally not been a target for PDT, with some exceptions such as intraperitoneal (colon, ovarian metastases) and intrapleural (mesothelioma) treatments. Clinical intent has ranged from cure of premalignant (dysplastic) and early-stage lesions through to salvage therapy and palliation. Trials have ranged from Phase I to Phase III, although relatively few large-scale randomized Phase III trials have been reported. The delivery of PS has been primarily intravenous for most solid tumors, topically for skin and cervical lesions and by instillation for bladder cancers. There have also been limited trials of intra-arterial embolization (Moore et al., 2008).

As mentioned above, light delivery systems have ranged from natural sunlight to lamps, LEDs (including “wearable” devices) and, particularly for endoscopic and interstitial light delivery, diode lasers. Depending on the particular tumor site and stage, treatment may be given on an out-patient or ambulatory basis, during endoscopy or intraoperatively, with the time between PS and light administration varying between minutes and days, depending on the PS pharmacokinetics.

The two-step activation and the various mechanisms-of-action of PDT confer a degree of “biochemical localization” of the PS to the site of disease, further enabled by the spatial localization of the activating light. This allows PDT to have a high anti-tumor activity with low damage to adjacent host tissue or surrounding organs. For example, skin malignancies are often treated with PDT with high efficacy and minimal damage or long-term cosmetic effect on the normal skin (Lui et al., 2004), and similar high therapeutic index is seen generally with PDT. This has led to implementation in salvage esophageal, head and neck, and other cancers recurring after definitive chemoradiation (Yano et al., 2012a; Vander Poorten et al., 2015). In these settings, PDT has demonstrated encouragingly high complete response rates (Kahaleh et al., 2008; Usuda et al., 2010; de Visscher et al., 2013).

Despite these multiple advantages, PDT has not been adopted as standard of care for many cancer indications, particularly in the United States. The National Comprehensive Cancer Network (NCCN) currently recommends considering PDT in basal cell carcinoma or cutaneous squamous cell carcinoma only when the disease is low-risk and superficial, and where surgery and radiotherapy is contraindicated (N.C.C. Network, 2020a; N.C.C. Network, 2020b). There are mentions in other settings such as for mesothelioma, where its use in combination with surgery is considered experimental (N.C.C. Network, 2020c). A summary of the recommended uses of PDT per NCCN guidelines is presented in Table 1. By comparison, other localized therapies such as RFA that were developed around the same time as PDT, have become front-line standard-of-care in US guidelines for multiple cancer sites (Friedman et al., 2004). A major challenge for PDT has been how to effectively treat deep-seated and larger solid tumors.

**TABLE 1:** NCCN recommendations for the use of PDT in standard-of-care settings across malignancies.

Cancer site	Recommended indication	PDT type	Level of evidence
Cutaneous basal cell carcinoma	Low-risk, superficial disease when surgery/radiotherapy not feasible	5-ALA/Photofrin with 570 nm–670 nm light source	Phase III randomized data versus cryotherapy Basset-Seguin et al. (2008)
Cutaneous squamous cell carcinoma	Precancerous lesions (Bowen’s disease, diffuse actinic keratosis)	Topical 5-ALA or methyl aminolevulinate (MAL) with red or green light source	Phase III randomized trial versus cryotherapy Morton et al. (2006)
Bladder	Diagnosis only	5-ALA with blue light to detect fluorescence	Meta-analysis Burger et al. (2013)
Mesothelioma	Alternative adjuvant therapy, not recommended over radiotherapy	Intravenous Photofrin with intracavitary 630 nm laser	Phase I/II series in conjunction with IMRT Du et al. (2010); Simone and Cengel (2014)
Central nervous system	Diagnosis only—intraoperative surgical adjunct	5-ALA with blue light to detect fluorescence	Phase III randomized trial against white-light microsurgery Stummer et al. (2006)
Non-small cell/small cell lung	Alternative palliative modality for malignant endobronchial obstruction or hemoptysis	Photofrin or chlorin PS with 630 nm laser	Phase II single arm study Friedberg et al. (2004)
Prostate	None, warrants further study	Padeliporfin with interstitial 753 nm laser	Phase III randomized trial against active surveillance in low-risk disease Azzouzi et al. (2017)

## The challenges of PDT in deep-seated and large solid tumors

Second- and third-generation PSs have been developed with significantly improved tumor specificity, targeting upregulated pathways in cancerous cells (Castano et al., 2004) and, thereby, enabling selective tumor destruction in the target volume with minimal off-target toxicity. This feature is especially advantageous in a theranostic approach, using fluorescence imaging of the PS to localize the target tissue with high precision and sensitivity (Daneshmand et al., 2018b).

**FIGURE 2:**
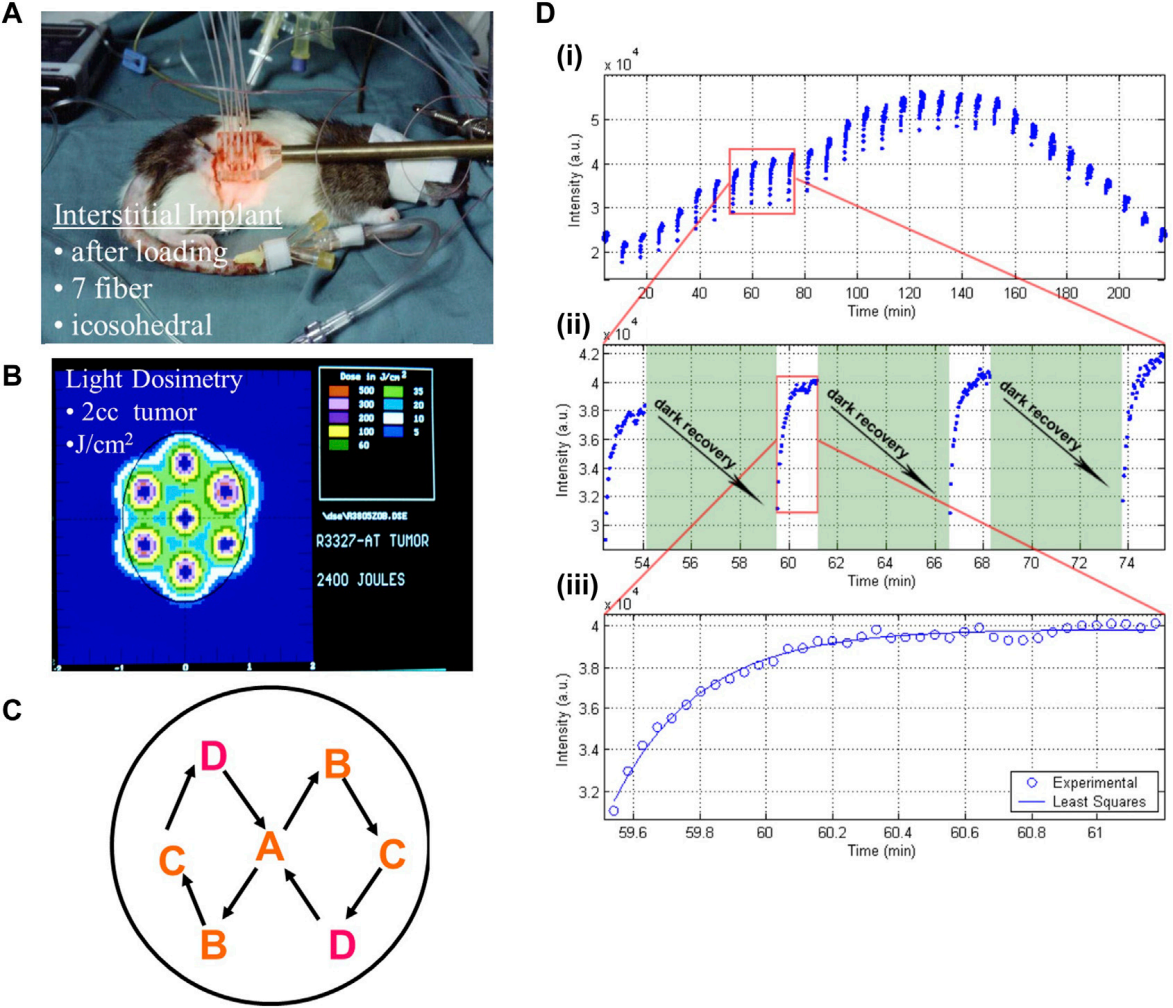
Methods of interstitial light delivery combined with spatial and temporal fractionation for optimizing PDT effect. A Dunning R3327 rat prostate cancer model was implanted with fiberoptics for light delivery in a standardized icosahedral layout **(A)**, adopted for ease of light dosimetry calculation and geometric expansion to larger tumors **(B)**. The fibers were activated sequentially in a specific geometric pattern **(C)** to allow fractionated therapy **(D)**. This deposits light (measured in arbitrary units, a.u., by the detecting light catheter/photodiode arrangement) in short bursts (each blue point) at regular intervals either continuously (top) or with pauses to allow for recovery of the photosensitizer (middle). This allows more homogenously and controls the rate of photobleaching and oxygen depletion in the treatment field, which leads to higher therapeutic yield. Image adapted from Xiao et al. (2007) with permission. a.u. = arbitrary units.

Nevertheless, for deep-seated and large solid tumors, delivering the activating light to and throughout the target volume remains challenging. As mentioned above, the limited depth penetrance of typical red/NIR activating light gives an effective treatment depth (or radius in the case of interstitial PDT: see below) of only 5–10 mm, depending on the tissue. The inhomogeneity in optical absorption and scattering of tumor tissue also makes it difficult to ensure that the complete target volume is fully treated (Dickey et al., 2004) and intratumoral heterogeneity of PS is also an issue. In addition, other factors, including the local light fluence rate, constant versus pulsed light delivery, variations in PS concentration in the target tissue, PS photobleaching (Dolmans et al., 2003) and low tumor oxygenation (baseline and PDT-induced) (Moore et al., 1993), can significantly affect the clinical outcomes (Xiao et al., 2007; van Straten et al., 2017). Light dosimetry modeling and fractionation techniques have been undertaken to address some of these challenges. For example, Figure 2 illustrates the use of standardized icosahedral light catheter placement along with switched pulse light delivery to deposit light homogenously, prevent photobleaching and rapid oxygen depletion.

The use of interstitial diffusing optical fibers for light delivery (iPDT) (Wilson and Patterson, 2008) has been employed clinically in multiple cancer sites, including prostate, pancreas, head and neck, brain and sites of metastases (Figure 2B) (Huang, 2005). However, Phase I, II and II clinical trials in these tumor groups have had varying levels of success. For example, Azzouzi et al. used the PS padaleporfin together with transperineal interstitial optical fibers and demonstrated significantly improved progression-free survival with minimal toxicity compared with active surveillance in low/intermediate-risk prostate cancer. iPDT with meta-tetra(hydroxyphenyl)chlorine (mTHPC) was employed successfully as a salvage treatment in a phase I/II trial of head & neck cancers that had recurred after chemoradiation (Lou et al., 2004a), achieving a 20% complete response rate, significant partial responses in almost all patients, some long-term survivors and no significant PS toxicities. Two patients did experience carotid rupture within 2 weeks of PDT due to disease invasion into the carotid, which speaks to the need for careful patient selection, given the strong and rapid necrotic tissue response to PDT (Lou et al., 2004a). Another phase I/II trial with verteporfin and MRI-guided iPDT in unresectable pancreatic cancer gave positive results in terms of tumor response and improved overall survival (Huggett et al., 2014), but the diameter of the treatment-induced tumor necrosis was variable, likely due to heterogeneities in light propagation, which speaks to the need for *in situ* dosimetry. The single interstitial fiber light delivery created up to a 12 mm diameter necrotic zone, so that treating larger tumors would have required the use of multiple sources. The latter approach has been most widely investigated in whole-organ targeted prostate cancer, where, for example, Davidson et al. (2009) used up to six cylindrically-diffusing fibers to treat recurrent cancer post radiation therapy and demonstrated a clear light-dose response threshold, above which 62% of the prostate was ablated, with minimal risk to normal tissues and ability to retreat if required. In metastatic disease, PDT has also proven effective for local control and symptom relief. For example, a recent Phase I trial for pathologic vertebral compression fractures in breast cancer patients with spinal metastases showed that PDT was safe, technically feasible and produced significant pain relief as an adjuvant to enable mechanical bone stabilization of the spine by destroying the space-occupying tumor mass (Fisher et al., 2019). Interstitial light delivery through vertobroplasty needles caused no short or long-term effects on the spinal cord that would have been of concern had the kyphoplasty been consolidated with palliative radiotherapy or stereotactic body radiotherapy (SBRT).

Despite positive clinical results with iPDT, accurately predicting the light dose distribution is significantly more susceptible to tissue heterogeneity than is the case with ionizing radiation, where soft tissues are uniformly “water-equivalent” (Wilson and Patterson, 2008). One strategy is to include interstitial light sensing fibers to monitor the distribution and fluence of the exciting light (Wilson and Patterson, 2008). This approach can provide feedback on calculated dosimetry, as well as real-time adjustment of light delivery during treatment to optimize standardization and therapeutic dose. The PS fluorescence can also be monitored in the same way. Clearly, this introduces an additional level of technical complexity to the treatment workflow and, to date, there has been a lack of consensus on the optimal approach: for example, using a few cylindrically-diffusing fibers or many point-diffusing sources to cover the target volume. Different models of light propagation in the target tissues have also been employed (Azzouzi et al., 2015). What does appear to be the case is that, at least for treating larger tumor volumes with curative intent, PDT treatment planning, energy delivery and dosimetry need to reach at least the same level of sophistication as has been achieved in radiation therapy, including brachytherapy with which it shares common features. The challenges are greater, however, because of PDT being a PS + light combination and tumor heterogeneity. Additional dosimetry tools, such as biophysical models and devices that integrate light, PS and oxygen levels (Wilson et al., 2003; Jarvi et al., 2012; Kosik et al., 2022), fluorescence monitoring of PS photobleaching (Sharwani and Alharbi, 2014), and direct measurement of ^1^O_2_ generation (Liang et al., 2012; Kim et al., 2016), show promise to address the challenge of achieving reliable individualized treatments.

## Addressing light penetrance in PDT

A first approach to effective PDT treatment of larger tumor volumes is to use PSs with strong light absorption and high ^1^O_2_ quantum yield at NIR wavelengths between about 700 and 850 nm, where there is the best trade-off between light penetration and PS activation efficiency (^1^O_2_ quantum yield). PSs such as chlorins, bacteriochlorins, phthalocyanines and hypocrellins can have quantum yields of 0.9 or greater (Yoon et al., 2013) and NIR activation can double the effective treatment depth (or radius for iPDT) in some tissues (Abrahamse and Hamblin, 2016) compared with the first-generation porphyrin-based Photofrin that is activated at 630 nm. These newer PSs come with their own pharmacokinetics and biodistribution/localization profiles (Menter et al., 1998) and some show a variable degree of skin photosensitivity (Dougherty et al., 1990). NIR activation also often leads to lower quantum yields of singlet oxygen, so requiring higher light doses and, without prolonging treatment time, also higher power input (up to 40 W/cm^2^) to compensate (Veeranarayanan et al., 2018). Furthermore, the accuracy of irradiating with NIR light at depth also becomes a challenge as potentially larger spot sizes are needed to achieve deeper penetration, which limits the precision achievable for the activating light source (Ash et al., 2017). A variant of NIR photosensitization is the use of upconverting nanoparticles that absorb multiple NIR photons to generate visible light to activate traditional PSs. However, these require high NIR intensity (approaching 10 W/cm^2^) and so may be limited by potential thermal damage to normal tissue (Cho et al., 2009; Qiu et al., 2018). These issues of accuracy and thermal loading can be improved with 2-photon PDT, where the PS is excited when it absorbs two different wavelengths, which also gives better spatial localization at the intersection of the two light beams (Castano et al., 2004). See Table 2 for a comparison of the modalities. Overall, however, moving to the near-infrared and improving PS specificity will not eliminate the need to use iPDT to treat larger, deep-seated and endoscopically inaccessible tumors.

**TABLE 2:** Summary of deep-penetrating PDT activation modalities.

Modality	PS	Depth of tissue penetrance	Disadvantages	Stage of development
NIR	Modified second and third generation	Up to 5 cm	Reduced ^1^O_2_ quantum yield	Mainly preclinical Li et al. (2020)
Two-photon	Nanoparticle-based	Up to 5 cm	Tissue heating over wider area	*In vitro*/*in vivo* studies Ogawa and Kobuke (2008), Han et al. (2020), Karges et al. (2020)
Upconverting nanoparticles	Nanoparticle-based	Up to 5 cm	Tissue heating	*In vitro*/early *in vivo* Tao et al. (2020)
Ultrasound	Modified chlorin-based	Up to 3 cm	Off target effects from high intensity ultrasound	*In vitro*/early *in vivo* Xu et al. (2021)

As mentioned above, a different strategy to treat deep/large tumors is to use ultrasound rather than light for PS activation. Sonodynamic therapy (SDT) has been demonstrated in various preclinical models (Yan et al., 2020) and a few small-scale clinical trials have shown potential benefit (Zha et al., 2023; D'Ammando et al., 2021). Most studies seem to use levels of PS and ultrasound that are close to their individual toxic doses, which is of concern. The ultimate safety and efficacy of SDT compared to PDT is still under investigation (Weber, 2018) and the underlying biophysical and biological mechanism of action are not well understood. SDT may also have problems with tissue heterogeneity and with effectively treating tumors deeper than a few cm without using more invasive techniques (Pfeiffer et al., 2015).

## Nanoparticle-mediated PDT systems

Beyond light delivery approaches, another way to maximize the therapeutic effect is to deliver the PS in a nanoparticle formulation, with or without active targeting, in so-called third-generation agents (Huang et al., 2012). These PDT nanoparticles aim to overcome biocompatibility issues with free PS, improve the kinetics and distribution characteristics and/or enable multimodal therapy. These issues can be particularly important with newer organic or inorganic PS constructs in order to trade biocompatibility and efficacy (Shim et al., 2022). Although some advantages can be achieved through conjugation of standard molecular PSs with antibodies, folate moieties or other small molecules, the majority of third-generation PDT approaches use nanoparticle systems (Dinakaran et al., 2023).

For nanoparticle-based PDT, the predominant constructs are with lipid nanoparticles (LNP) and their derivatives. LNPs here refer to a family of lipid-based nanoparticles such as micellar structures (single layer phospholipid with hydrophobic interior), liposomes (bi-layer phospholipids with physiologic interior environment), solid LNPs (admixture of lipids along with payload) and similar carriers. These are an attractive carrier system for PS because of the extensive research that has made them stable, biocompatible, have favorable distribution properties and drug release kinetics, provide passive targeting through the enhanced permeability and retention (EPR) effect or allow active targeting via aptamers, allow simultaneous co-delivery of other cancer drugs, and make even natively toxic drugs feasible to use in humans (Avci et al., 2014; Dinakaran et al., 2023). Beyond this obvious advantage, further development of LNP-based PDT agents has resulted in some new strategies in leveraging nanoparticles for PDT.

One optically active LNP agent was first reported by Lovell et al. (2011) with self-assembling porphyrin-lipid nanoparticles (Porphysomes) that exhibited high biocompatibility, minimal systemic toxicity (up to 1,000 mg/kg in mice), photothermal activation in the intact state and photodynamic activation when dissociated after cell uptake (Guidolin et al., 2021). Other iterations of Porphysomes have demonstrated theranostic capabilities with fluorescence and photoacoustic imaging (Muhanna et al., 2015). These examples show that LNP systems for PDT can not only gives the expected biocompatibility and distribution benefits as with chemotherapeutic payloads but can also present opportunities for unique new applications. Some drawbacks can still exist, however, namely, challenges in synthesis to achieve appropriate characteristics of size, polydispersity and encapsulation efficiency (Hou et al., 2021). Another issue is the tendency for LNP encapsulated PS to self-quench, which is why systems such as Porphysomes are not photodynamically active until dissociated into porphyrin-lipid monomers *in vitro* or *in vivo* (Muhanna et al., 2015).

Beyond LNPs, the next most common nanocarrier platform for PSs is polymeric nanoparticles such as poly-lactic-co-glycolic acid (PLGA). PLGA also has a track record of preclinical and clinical use to carry chemotherapeutic agents, albeit less developed than LNPs (Dinakaran et al., 2023). For the PLGA may have some advantages over LNP for photosensitizer delivery. PLGA is particularly effective at encapsulating strongly hydrophobic drugs such as PSs derived from porphyrins, hypocrellins and pthalocyanates (Kou et al., 2017). PLGA also typically has higher stability and slower drug-release kinetics than LNPs due to thicker walls, increased rigidity and lower permeability (Avci et al., 2014). This may be a hindrance for achieving slow-release with chemotherapeutics but can be beneficial for PDT where the PS can remain stable and in tissue for longer before light activation at the site of disease. These factors have helped PLGA achieve success in many preclinical PDT studies, such as with hypericin-loaded PLGA that has shown higher photoactivity and therapeutic index in ovarian cancer cells compared to PS alone (Zeisser-Labouèbe et al., 2006).

In addition to the dominant LNP and polymeric nanoparticle platforms, many other PS-nanoparticle constructs have been investigated preclinically, including inorganic systems such as gold nanoclusters (Maiolino et al., 2015), nanosilica (Karges et al., 2021), dendrimers (Avci et al., 2014) and fullerenes (Hamblin, 2018). While showing some favorable characteristics, currently none possess the balance of biocompatibility, biodistribution, toxicity and clinical implementation data enjoyed by LNPs and polymeric nanoparticles. The application of nanoparticles to PDT is still actively under research and one particularly exciting possibility is light-independent activation, such as with ionizing radiation (Dinakaran et al., 2020).

## Nanoparticle augmentation of radiotherapy

As a widely used modality, significant investment has been made to advance radiotherapy and maximize the therapeutic ration of anti-cancer effect-to-toxicity. This has included refinement of radiobiology-based strategies in the 1970s–2000s (Kirsch et al., 2018) and the increasing precision of radiation delivery (Pacelli et al., 2019). A new wave of interest in combination therapies, such as chemotherapy or targeted biologics plus immunotherapy has met with success in some, but not all, cancer types (Patrick et al., 2017; Nakhoda and Olszanski, 2020; Gong et al., 2021; Lin et al., 2022; Lu et al., 2022). Further advancements in combining radiotherapy with systemic agents are still underway with more bespoke, radiation-specific agents, and a variety of nanoparticle-based agents significantly contribute to this.

Nanoparticle-mediated radiosensitizers can be split into two groups: metallic and non-metallic. The best known metallic radiosensitizers are the high-Z materials such as gold nanoparticles (Kwatra et al., 2013). These are intended to increase the physical X-ray dose deposition in the target tumor and can enhance the efficacy by typically ∼2-fold (Kwatra et al., 2013). The mechanism is higher energy conversion through photoelectric effect and Compton effects, pair production and Auger electron generation (Kwatra et al., 2013). The dose enhancement is particularly pronounced below about 1 MeV photon energy, mediated by the photoelectric effect, increasing the probability of photon interaction and electron production by the Z^3^ (Almahwasi, 2016). The most clinically successful of these metallic nanoparticles is hafnium oxide, which effectively doubled complete responses in advanced soft tissue sarcoma, with minimal additional toxicity, as shown in Phase II/III clinical trials (Bonvalot et al., 2019). However, this required intratumoral injection, which limits its homogeneous intratumoral distribution and the clinical practicality for many sites (Holback and Yeo, 2011).

Efforts to use newer metallic nanoparticles designs aim to address some of these practical issues and further improve the efficacy. A common approach is by “decorating” the nanoparticles with moieties aimed at increasing biocompatibility or enhancing tumor-specific uptake. For example, coating metal nanoparticles in polyethylene glycol (PEG) facilitates intravenous injection, enhancing the pharmacologic properties and tumor uptake via the aforementioned EPR effect (Chattopadhyay et al., 2013). Other active-targeting strategies, e.g., conjugation with targeting antibodies against Human Epidermal growth factor Recepter-2 (HER-2) tumor antigens, can improve tumor accumulation and enhance treatment efficacy. Thus, HER-2 conjugated gold nanoparticles to treat breast cancer increase tumor cytotoxicity by 50% over untargeted radiosensitization alone (Chattopadhyay et al., 2013).

Although quite successfully preclinically, metal-based nanoparticles have limitations in clinical use due to the different photon energies used clinical radiotherapies. Typically, clinical linear accelerators produce photons with 6 MV or greater energy, where the dominant Compton effect is only weakly Z-dependent, greatly reducing the dose enhancement seen preclinically using lower energy irradiators where the photoelectric effect dominates (Mesbahi, 2010).

Alternative mechanisms of non-metallic nanoparticle radiosensitization have been explored that are not susceptible to this limitation, such as polymeric nanoparticles that deliver established chemotherapy-based radiosensitizers. Specific examples include Genexol-PM, paclitaxel-based nanoparticles that produced comparable radiosensitization in xenograft tumors models as paclitaxel alone but with less toxicity (Werner et al., 2013). Similar results have also been seen with free vs. nanoparticle-delivered doxorubicin (Werner et al., 2011). Another class of non-metallic radiosensitizers produces direct cellular toxicity under irradiation. Thus, superparamagnetic iron oxide nanoparticles (SPION) can amplify the effectiveness of the ROS generated by ionizing radiation, increasing the cytotoxicity by 2–3 fold (Huang et al., 2013; Kwatra et al., 2013). SPIONs have the added benefit of being visible on MRI and can be actively targeted using external magnetic fields. Other agents that are earlier in development, such as silica-based nanoparticles and fullerene structures, can produce direct cell membrane and organelle damage during irradiation (Kwatra et al., 2013).

Beyond these approaches, future nanotechnology-based strategies included nanoparticlized oxygen mimetics that radiosensitize through irreparable fixation of radiation-mediated DNA damage, but significantly reduce the toxicity of typical oxygen mimetics (Meng et al., 2018). An evolving field is the use of oncologic therapies with cytotoxic mechanisms that are complementary to radiation effects, with ROS-based agents being of particular interest (Howard et al., 2020). In this respect the mechanisms of PDT cell-kill that have been known for many years are particularly complementary to radiation (Stewart et al., 1998b). Advances in nanotechnology in the last 20 two decades has allowed realization of radiation-PDT combinations that exploit this complementarity (Viswanath and Won, 2022).

## Potential radiotherapy-PDT integration

Some of the advantages and limitations of PDT are in part complementary to those of radiotherapy, and there is a degree of shared knowledge and understanding of some of the technical and practical issues, for example, around delivery to the tumor target, avoidance of collateral damage to normal tissues, and EM energy propagation and dosimetry (see Table 3). Beyond such parallels, there are emerging potential roles for PDT to advance radiation oncology and *vice versa.*

**TABLE 3:** Comparison of advantages and limitations of photodynamic therapy (PDT) and radiotherapy (RT) as currently practiced.

	Advantages		Limitations	
	PDT	RT	PDT	RT
Physical	Dual-selectivity (PS + light)	Deep tissue penetration	Limited light penetration	Normal tissue dose from beam entry/exit
	Intratumoral light delivery	Homogeneous radiation attenuation in soft tissues	High optical heterogeneity	Primarily single mechanism effect (DNA damage)
	Endoscopic and intraoperative delivery	High technical and dosimetric precision	Complex dosimetry and photobleaching	Radiation safety, logistics
Biological	Rapid cell-kill and tumor response	Differential sensitivity of tumors (death) versus normal tissues (repair)	Heterogeneity of drug pharmacokinetics and localization Energy inefficient—high input energies typically used. Requires optimal photosensitizer-light time interval	Intrinsic and treatment-induced radio-resistance
	Multiple cytotoxic pathways and tumor targets	Energy efficient: DNA damage amplifies biological effect		Mixed immune effects of immune-priming and immune-dampening
	Oxygen-independent photosensitizers available	Well defined (stochastic) dose and effect relationships		Reduced efficacy in hypoxic tumors
	Minimal treatment-induced resistance	Extensively modeled biological response		Risk of radiation-induced malignancy
	Potent Immune upregulation			
Clinical	Minimal systemic or regional toxicity	All tumor sites accessible using same technological platform	Implementation requires various interventional clinical specialists	Cumulative dose limiting normal tissue damage restricts repeated treatment
	Repeatable—no “lifetime” maximum dose	Few patient or disease-related contraindications	Deep-seated and large tumors challenging	Acute and long-term toxicities
	Works in radioresistant tumors and in post-RT/chemotherapy/surgery recurrence	Proven curative and palliative applications	Skin photosensitivity with some PSs	Large and costly infrastructure
	Relatively low cost: point-of care delivery	Predictable side-effects by tissue type and dose delivered	Difficult to standardize treatment delivery due to light and PS inhomogeneities in tumor	Multi-fraction treatment can be logistically challenging for patients and introduce inter-fraction setup variability
		Normal tissue structure and nerve sparing		

The most direct approach is simply to combine PDT and radiotherapy as distinct modalities, each optimized separately in individual patients in whom the single modalities may be sub-curative, because of inadequate tumor response and/or dose-limiting normal tissue toxicities. Importantly, the primary mechanisms of (tumor) cell death are distinct between the modalities, with radiotherapy (external beam or brachytherapy) being mediated by DNA damage leading to proliferative cell death or cytostasis (Baskar et al., 2014), and PDT acting via through ROS-mediated damage in extra-nuclear cell structures (e.g., mitochondria) to tumor cells and/or vascular endothelial cells, and also possibly leading to immune modulation. These complementary mechanisms of action may synergize the anti-tumor efficacy without greatly increasing collateral toxicity. Most preclinical combination studies have demonstrated at least additive effects (Allman et al., 2000; Bulin et al., 2019) and some have demonstrated possible synergy: e.g., MCF-7 breast cancer cells treated *in vitro* with X-rays and PDT showed a much greater decrease in cell viability than either treatment alone, even when doses were de-escalated for both modalities (Santos et al., 2019). Potential synergy was also seen by Nakano et al. (2011) in treating patients with recurrent Bowen’s Disease with PDT and electron radiotherapy, where complete response rates of 80%–100% were achieved with no additional normal tissue toxicity. Similarly encouraging results have also been seen with endobronchial tumors, sarcomas and esophageal cancers (Sanfilippo et al., 2001; Freitag et al., 2004; Kusuzaki et al., 2005).

Even without mechanistic synergy, an important characteristic of PDT is that it can be safely and effectively used either before or after radiotherapy, without causing excess toxicity or inducing resistance. Thus, PDT has been used in several patient cohorts with locally-recurrent disease following radiotherapy (Lou et al., 2004b; Wildeman et al., 2009; Yano et al., 2012b; Gangloff et al., 2012). The advantage is that, even in patients whose normal tissues cannot tolerate more radiation treatment, PDT can be given and produce good responses without added toxicity: in part, this works because (due to the limited penetration of light and the use of interstitial light delivery) there is little spread of PDT damage beyond the target tumor volume and in part because of the different cellular targets, so that even cells that are intrinsically radioresistant can be killed by PDT, as shown definitively *in vitro* (Stewart et al., 1998a; Oniszczuk et al., 2016). Radiation can also be delivered safely post PDT treatment, an example being in glioma where PDT is used immediately post-surgery to selectively kill surviving mesoscopic disease in the vicinity of the surgical bed and chemoradiation is then used to kill residual microscopic and disseminated disease (Rigual et al., 2013; Schipmann et al., 2021).

An emerging strategy is to directly combine radiotherapy with PDT by using X-rays or radionuclides as the energy source to activate the PS, thereby accessing deep-seated and large tumors non-invasively. This technique is known variously as “radioPDT,” “X-ray PDT,” “radiodynamic therapy” and others (Ren et al., 2018; Cline et al., 2019; Dinakaran et al., 2020). One exemplary technique would be the use high-energy X-rays as delivered, for example, in clinical radiotherapy using a LINAC but at a low, well-tolerated radiation dose (typically, <10 Gy). The preclinical studies of this technique have frequently exploited different nanoparticle formulations, either as X-ray activatable materials and/or as delivery vehicles for molecular photosensitizers (Dinakaran et al., 2023). Several specific molecular radioPDT agents are also being developed (Larue et al., 2018), as well as an emerging approach is the use of nanoclusters, whose small number (∼10–30) of gold or silver atoms confers molecule-like quantum energy levels that result in singlet oxygen generation under X-ray exposure (van de Looij et al., 2022).

Three different and possibly complementary physical activation pathways have been investigated: direct photosensitizer activation mediated by high-energy secondary electrons, radioscintillation light emitted from nanocrystals co-delivered with the photosensitizer within, typically polymeric, nanoparticles, and activation by the Cherenkov light generated in tissue by high-energy (>MeV) secondary electrons. For any of these nano materials and activation pathways, and depending on the PS localization, the subsequent photophysics, photochemistry and photobiology should be similar to conventional PDT using external light sources.

The potential for X-rays to elicit photodynamic response was shown first by Chen et al. (2006). X-rays induced luminescence from nanoscintillators and excited a photosensitizer *via* Förster resonance energy transfer (FRET) (Chen and Zhang, 2006; Retif et al., 2015). This was significantly more cytotoxicity in tumor cells *in vitro* than radiation alone and was mediated not only by DNA damage but also through cytoplasmic organelle stress. Multiple subsequent studies, particularly over the last 10 years, have evolved novel iterations of radioPDT nanoparticles aimed at increasing the therapeutic yield, optimizing the physical characteristic and/or targeting of specific cellular structures (Retif et al., 2015; Clement et al., 2016). If this can be successfully translated to the clinical setting, radioPDT could address shortcomings of both conventional PDT and radiotherapy, since it combines the high tissue penetrance, precise targeting and accurate dosimetry of modern radiotherapy with the superior tumor-control capability (both cytotoxicity and secondary immune upregulation) and low toxicity of PDT. In addition, tumor targeting of the PS, with or without the use of nanoparticles, could further increase the therapeutic index (Moore et al., 2008).

Despite promising results, several significant challenges remain for clinical translation. The first is the need to increase the efficiency, i.e., the cell kill per Gy radiation dose: although the principle of radioPDT has been well demonstrated *in vitro*, the level of tumor cell kill at sub-10 Gy dose is not large, so that many *in vivo* studies have resorted to intra-tumoral injection of the photosensitizer/nanoparticles to achieve a useful degree of tumor control. There are two primary strategies being pursued to address this limitation, based on improving either the radiation-optical physics and/or the radio-photobiology. The first requires consideration of the multiple steps in the activation pathway. For example, in using scintillation activation, this includes i) increasing the X-ray cross-section and quantum yield of light emission by the scintillation nanocrystals, ii) maximizing the spectral overlap and minimizing the separation of the scintillator and photosensitizer in order to maximum FRET, which is more efficient than 2-step scintillator luminescence emission and its subsequent absorption by the PS, and iii) increasing the ^1^O_2_ quantum yield of the PS. (Chen and Zhang, 2006; Clement et al., 2016). Further refinements of the nanoscintillator used can also lead to tuned characteristics with the photosensitizer, such as persistently luminescent nanoparticles (Liu et al., 2021) that can build on the light dosimetry work (Figure 2) to prevent quenching of higher potency photosensitizers (Xiao et al., 2007). Similarly, in Cherenkov-mediated radioPDT the main requirement is to maximize the spectral overlap of the photosensitizer with that of the Cherenkov light after it has propagated through the tissue, which is strongly tissue- and wavelength-dependent given that the Cherenkov spectrum is mainly in the UV and short-wavelength visible range.

The other option of increasing the biological efficiency of radioPDT, requires that a given cellular concentration of ^1^O_2_ is maximally cytotoxic, either directly in tumor cells or indirectly through vascular endothelial damage. A major factor in determining this intrinsic sensitivity is the subcellular localization of the PS. In particular, several studies with conventional (i.e., external light-activated) PDT have shown that the cytotoxicity can be increased by 3 orders-of-magnitude or more if the PS is localized in the cell nucleus rather than in cytosolic organelles, so that the resulting damage is primarily to DNA (Yu et al., 2016; Niculescu and Grumezescu, 2021; Long et al., 2022). This could be done by suitable targeting of the PS (and, if used, the nanoscintillator), for example, using TAT peptides. Note, however, that purely DNA damage-mediated radioPDT would likely result in loss of the advantageous secondary immune effects of PDT: this is probably not a major concern, since nuclear targeting is far from 100% efficient. DNA damage from radioPDT over and above the “background” level due to the ionizing radiation itself has been demonstrated using the clinical photosensitizer verteporfin (Clement et al., 2021). It was also shown using Cherenkov-light activation of psoralens that are routinely used in psoralen-ultraviolet A (PUVA) treatment of benign skin diseases (Vangipuram and Feldman, 2016) and have a degree of intrinsic (i.e., passive) nuclear localization. In one study, Radiation Enhanced with Cherenkov photo-Activation (RECA) demonstrated ∼10%–20% improvement in *in vitro* cytotoxicity over radiation alone (Yoon et al., 2018) and resulted in tumor growth inhibition *in vivo* (Oldham et al., 2016).

The requirement of achieving a large “photodynamic enhancement factor,” i.e., increased cell kill for a given radiation dose, to use radioPDT as a stand-alone treatment, is substantially reduced by using radioPDT as a novel form of radiosensitization for conventional fractionated radiotherapy, operating through novel photophysical processes rather than through radiochemistry as in traditional agents (Wang et al., 2016; Berry and Fan, 2021; Cockshott, 2021; Gong et al., 2021). This has been demonstrated using X-ray Cherenkov activation of psoralens, where ∼1%/Gy decrease in survival of B16 melanoma cells was achieved. While an important proof-of-principle, this level of sensitization would not translate into a biologically significant increase in efficacy over typical fractionated doses (ex: 60 Gy over 30 fractions), whereas about 10%/Gy decrease in cell survival gives 2–3 logs of cell kill and would be clinically significant. Interesting, in addition to the increased cell kill, psoralens also upregulated the expression of upregulated Major Histocompatibility Complex-I (MHC-I) up to 450% (Yoon et al., 2018). It will be interesting to see if this increase in immunogenic markers also confers an immune response through the non-nuclear component of the PS localization.

The second major challenge for radioPDT is that many of the proposed agents utilize novel, often exotic, structures and components that raise concerns for biocompatibility, *in vivo* toxicity, tumor localization and pharmacokinetics. In part, these factors arise from the nanoparticulate formulation itself as used in some studies, while scintillation-mediated radioPDT has the added factor of potentially toxic rare-earth-based nanocrystals (e.g., CeF_3_). Addition of antibodies, peptides or other tumor cell- or organelle-targeting moieties adds further complexity to the formulation (Clement et al., 2018; Deng et al., 2020; Clement et al., 2021).

Another important factor in radioPDT is the dependence on molecular oxygen that both PDT and radiotherapy share. In the sequential PDT-radiation or radiation-PDT approaches considered above, there is an opportunity for a degree of tumor re-oxygenation to occur between modalities, thereby increasing the efficacy, which is not the case with stand-alone radioPDT. Of note, due to the low rate of energy deposition rate used radiotherapy vs. PDT, it is unlikely that the photochemical depletion of molecular oxygen that is seen with conventional PDT at high light fluence rates will occur in radioPDT. An exception may be if FLASH irradiation is used, where the dose deposition rate is orders of magnitude higher (Matuszak et al., 2022). Many solid tumors contain a subset of hypoxic cells with pO_2_ <4% (Milosevic et al., 2012), which is in the range of the 50% sensitization point for oxygen enhancement factors in mammalian cell kill for PDT and radiotherapy of ∼0.5%–2% (Moore et al., 2007) and ∼3%–4% (Grimes et al., 2017), respectively. Hence, a detailed understanding of the performance of radioPDT under low pO_2_ conditions is needed in order to minimize the potential negative impact of tumor hypoxia. To date, only one study has systematically evaluated this: the cytotoxic effect was indeed reduced under highly hypoxic conditions but was preserved, at least *in vitro*, for pO_2_ >1% by optimizing the concentration of the PEG-PLGA encapsulated LaF_3_:Ce^3+^ nanoscintillator and PpIX photosensitizer nanoparticles and the radiation dose (Dinakaran et al., 2019); e.g., the cytotoxicity benefit of radioPDT over radiation alone was as high as 50% even under hypoxic conditions. Other groups have demonstrated the use of high quantum efficiency metallo-organic photosensitizer complexes (Azad et al., 2023) that are efficient in normoxia and hypoxia as well as oxygen-independent inorganic photosensitizers such as psolarens (Pathak, 1984; Zhang et al., 2015a), although delivery of these to poorly vascularized tumors may still be limiting. Use of the Type I reaction pathway, which relies on electron transfer to generate oxy and hydroxy radicals, is a further potential strategy to mitigate the effect of tumor hypoxic (Zhang et al., 2015b) and has demonstrated efficacy both *in vitro* and *in vivo* at physiologic tumor environments, including hypoxia (Dinakaran et al., 2019).

Notwithstanding these challenges, the potential theranostic utility, especially through the use of multifunctional nanoformulations, may facilitate clinical translation and impact of radioPDT. To date, much of the focus of theranostics, i.e., image-guided therapies, has been in radionuclide-based systems (Turner, 2018), with other operational modes such as photoacoustic, fluorescence and MRI-based systems emerging rapidly (Wang et al., 2020; Brito et al., 2021; Sarbadhikary et al., 2021). The fluorescence emission of the photosensitizer during radioPDT irradiation could be used in treatment planning and dosimetry, while theranostic radioPDT agents may be of value also in image-guided radiation therapy (IGRT). For example, some proposed radioPDT nanoparticles also show X-ray CT (or MRI) contrast (Chen et al., 2017b; Dinakaran et al., 2018; Xu et al., 2019) which would allow tracking them using, e.g., onboard imaging devices built into a LINAC. This would aid in ensuring adequate tumor uptake and optimal intra-tumoral localization of the radioPDT agent and to compensate for variations in pharmacokinetics. In particular, if the concentration in the tumor can tracked and quantified, then, combined with accurate radiation dosimetry, the effective radioPDT therapeutic yield could be predicted. This would enable better standardization of treatments and reduce variability arising from pharmacokinetic differences between patients or even in the same patient over successive fractions, thereby improving the overall quality and outcome of IGRT. Several radioPDT variants leverage nanotechnology to allow for such theranostic capabilities and there are many other targeting, biocompatibility and multimodal treatment strategies that could be developed (Chen et al., 2017c; Dinakaran et al., 2020; Clement et al., 2021; He et al., 2022).

## Conclusion

PDT has potential benefits in oncology as an alternative stand-alone or adjuvant treatment modality. While many preclinical studies and a variety of clinical trials have demonstrated long-term tumor control, manageable short-term toxicity and virtually no long-term toxicity, PDT has not yet gained widespread clinical adoption in oncology beyond relatively limited specific applications. We have argued above that significant factors in this are the technical complexity and variable responses, particularly in treating large tumor masses and deep-seated tumors that are not endoscopically accessible. While there is certainly benefit in applying some of the treatment planning and dosimetry concepts developed for radiotherapy, and these are being implemented in some current clinical trials, the fundamental challenge is the limited and strong tissue- and wavelength-dependence of the penetration of visible and, albeit to a lesser extent, near-infrared light in tissues. On the other hand, there are distinct and clinically-significant biological advantages of PDT’s mechanism of action.

The interplay between PDT and radiation therapy to maximize the therapeutic efficacy and minimise side effects has been underexploited to date, despite the well-established absence of contraindications between the two modalities and their complementary mechanisms of action that yield potential synergism. A clear example is the efficacy of PDT in radioresistant tumors that, as described above, PDT can effectively salvage. This is a missed opportunity for both communities.

Closer integration may result from overlapping interest in radioPDT, used either as a stand-alone “acute” modality (possibly in combination with conventional radiation treatment) or as a novel form of radiation sensitization. Like conventional PDT itself (Akasov et al., 2019), its mechanisms-of-action through ROS generation can be tailored to maximize either direct or, via microvascular shutdown, indirect tumor cell death. In either case, immune upregulation should confer an additional level of local tumor control, with possible impact also on tumor progression and risk of metastatic spread. This field is still at an early stage of preclinical development, so that there are many avenues to be explored. The complementary basic and clinical perspectives, and over a century of experience in both optical and radiation biosciences, could accelerate this development and ensure that ultimately the modality will be translated into oncologic practice. Through radioPDT, the ability of advanced radiotherapy techniques to target lesions in the body can be interfaced with PDT’s biological effectiveness. This can advance the field of radiation oncology by providing greater therapeutic effect even in radioresistant tumors, without additional significant toxicity.
